# Isotypic Mn^II^ and Fe^II^ binuclear complexes of the ligand 5,6-bis­(pyridin-2-yl)-pyrazine-2,3-di­carb­oxy­lic acid

**DOI:** 10.1107/S2056989016014055

**Published:** 2016-09-09

**Authors:** Monserrat Alfonso, Helen Stoeckli-Evans

**Affiliations:** a Institute of Chemistry, University of Neuchâtel, Av Bellevaux 51, CH-2000 Neuchâtel, Switzerland; b Institute of Physics, University of Neuchâtel, rue Emile-Argand 11, CH-2000 Neuchâtel, Switzerland

**Keywords:** crystal structure, isotypism, cage-like binuclear complex, manganese(II), iron(II), 5,6-bis­(pyridin-2-yl)pyrazine-2,3-di­carb­oxy­lic acid, hydrogen-bonded framework

## Abstract

The title isotypic complexes, bis­[μ-5,6-bis­(pyridin-2-yl)pyrazine-2,3-di­carboxylato]-κ^4^ *N* ^1^,*O* ^2^,*N* ^6^:*O* ^3^;κ^4^ *O* ^3^:*N* ^1^,*O* ^2^,*N* ^6^-bis­[di­aqua­manganese(II)] tetra­hydrate, [Mn_2_(C_16_H_8_N_4_O_4_)_2_(H_2_O)_4_]·4H_2_O, (I), and bis­[μ-5,6-bis­(pyridin-2-yl)pyrazine-2,3-di­carboxyl­ato]-κ^4^ *N* ^1^,*O* ^2^,*N* ^6^:*O* ^3^;κ^4^ *O* ^3^:*N* ^1^,*O* ^2^,*N* ^6^-bis­[di­aqua­iron(II)] tetrahydrate, [Fe_2_(C_16_H_8_N_4_O_4_)_2_(H_2_O)_4_]·4H_2_O, (II), are, respectively, the mangan­ese(II) and iron(II) complexes of the ligand 5,6-bis­(pyridin-2-yl)-pyrazine-2,3-di­carb­oxy­lic acid. The complete mol­ecule of each complex is generated by inversion symmetry. Each metal ion is coordinated by a pyrazine N atom, a pyridine N atom, two carboxyl­ate O atoms, one of which is bridging, and two water O atoms. The metal atoms have *M*N_2_O_4_ coordination geometries and the complexes have a cage-like structure. In the crystals of both compounds, the complexes are linked by O—H⋯O and O—H⋯N hydrogen bonds involving the coordinating water mol­ecules, forming chains along [100]. These chains are linked by O—H⋯O hydrogen bonds involving the non-coordinating water mol­ecules, forming layers parallel to (011). The layers are linked by pairs of C—H⋯O hydrogen bonds and offset π–π inter­actions, so forming a hydrogen-bonded three-dimensional framework.

## Abstract 2

The reaction of manganese dichloride and iron dichloride with the ligand 5,6-bis­(pyridin-2-yl)pyrazine-2,3-di­carb­oxy­lic acid leads to the formation of isotypic binuclear complexes which have a cage-like structure.

## Chemical context   

The syntheses and crystal structures of the ligand 5,6-bis(pyridin-2-yl)pyrazine-2,3-di­carb­oxy­lic acid (**H_2_L**) and three different salts, have been described by Alfonso *et al.* (2001 ▸), and it was noted that the ligand crystallizes as a zwitterion in all four compounds. The reaction of **H_2_L** with CuBr_2_ (ratio 1:2) led to the formation of a one-dimensional coordination polymer. On exposure to air, this compound loses the solvent of crystallization and four water mol­ecules, transforming into a polymeric two-dimensional network structure (Neels *et al.*, 2003 ▸). In both cases, there are two crystallographically independent fivefold-coordinated copper atoms present, each having an almost perfect square-pyramidal geometry. Recently, we have reported on the crystal structure of the cadmium dichloride complex of ligand **H_2_L**, which is a two-dimensional coordination polymer (Alfonso & Stoeckli-Evans, 2016 ▸). Herein, we describe the syntheses and crystal structures of the title isotypic binuclear complexes, (I) and (II), formed by the reaction of **H_2_L** with, respectively, MnCl_2_ and FeCl_2_.
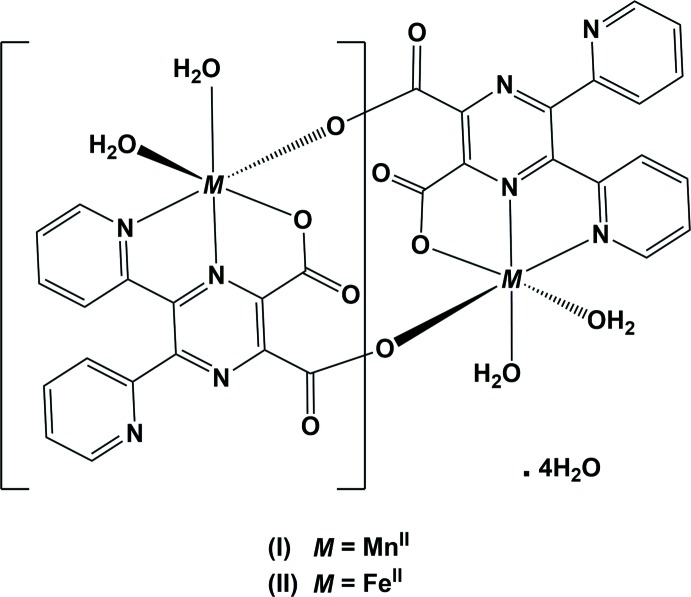


## Structural commentary   

**Figure 1:**
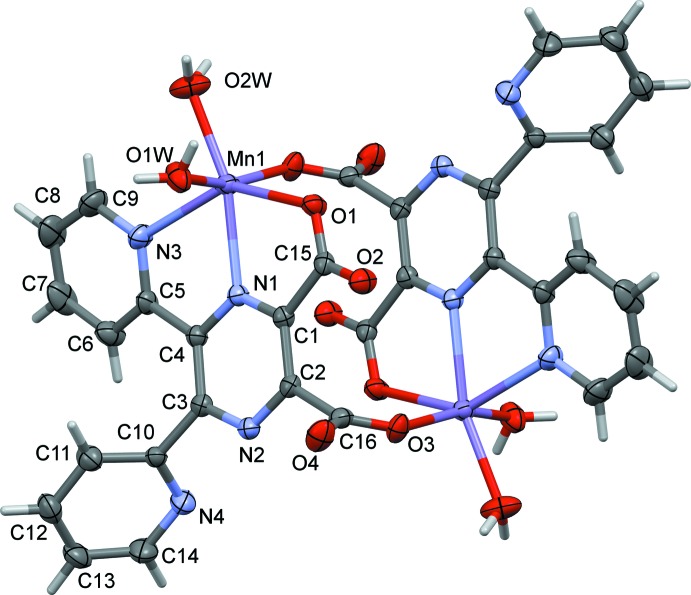
A view of the mol­ecular structure of compound (I), with atom labelling. Unlabelled atoms are related to the labelled atoms by inversion symmetry (−*x* + 2, −*y* + 2, −*z* + 2). Displacement ellipsoids are drawn at the 50% probability level. The solvate water molecules have been omitted for clarity.

**Figure 3:**
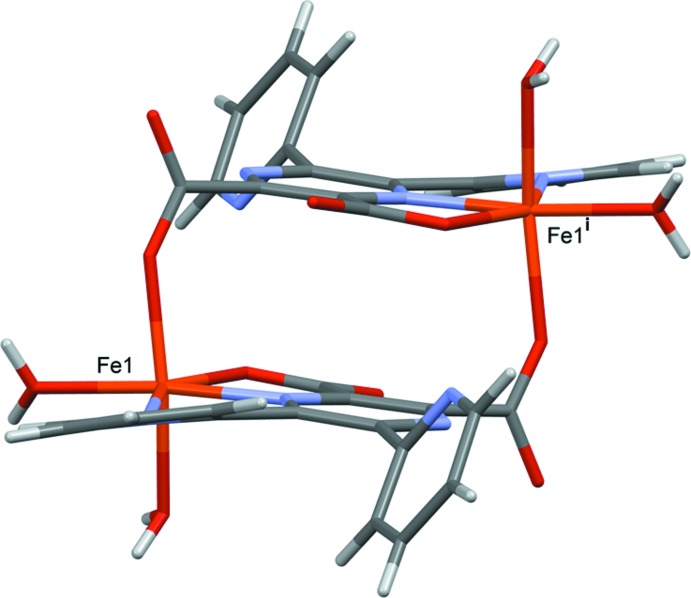
A view of the mol­ecular structure of compound (II), illustrating the cage-like form of the complexes [symmetry code: (i) −*x* + 2, −*y* + 2, −*z* + 2].

The complete mol­ecules of complexes (I) and (II) are generated by inversion symmetry, as shown in Figs. 1 ▸ and 2 ▸, respectively. The metal atoms are sixfold coordinated by one pyrazine N atom (N1), one pyridine N atom (N3), two water O atoms (O1*W* and O2*W*), and by two carboxyl­ate O atoms, O1 and O3^i^ [symmetry code: (i) −*x* + 2, −*y* + 2, −*z* + 2]. Hence, the ligand coordinates to the metal atoms in a tridentate (*N,N,O*) and a monodentate (*O*) manner. Atom O3 is bridg­ing, so leading to the formation of a cage-like complex situated about a centre of inversion; illustrated in Fig. 3 ▸ for the Fe^II^ complex, (II). The metal–metal distances are Mn1⋯Mn1^i^ *ca* 6.58 Å, while the Fe1⋯Fe1^i^ distance is *ca* 6.50 Å. Selected bond lengths and angles for compounds (I) and (II), are given in Tables 1 ▸ and 2 ▸, respectively.

In complex (I), it can be seen from the carboxyl­ate C—O bond lengths [C15—O1 and C15—O2 are 1.257 (4) and 1.243 (4) Å, respectively, and C16—O3 and C16—O4 are 1.254 (4) and 1.239 (4) Å, respectively], that the negative charge is distributed over the O–C–O groups (Table 1 ▸). The Mn—N_pyrazine_, Mn1—N1, bond length is 2.242 (3) Å, which is shorter than the Mn—N_pyridine_, Mn1—N3, bond length of 2.311 (3) Å. The Mn1—O_water_ bond lengths [2.141 (3) and 2.148 (3) Å] are similar to the Mn—O_carboxyl­ate_, Mn1—O3^i^, bond length of 2.139 (2) Å, while distance Mn1—O1 is longer at 2.228 (2) Å.

In complex (II), the carboxyl­ate C—O distances [C15—O1 and C15—O2 are 1.271 (4) and 1.229 (4) Å, respectively, and C16—O3 and C16—O4 are 1.251 (3) and 1.240 (4) Å, respectively], indicate that the negative charge is centred on atom O1 for carboxyl­ate O1–C15–O2, while for carboxyl­ate O3–C16–O4 is appears to be distributed over the O–C–O group (Table 2 ▸). This situation is similar to that observed for the coordinating carboxyl­ate groups in the Cd^II^ two-dimensional coordination polymer involving ligand **H_2_L**, mentioned above. The Fe—N_pyrazine_ bond length, Fe1—N1, is 2.126 (2) Å, which is slightly shorter than the Fe—N_pyridine_, Fe1—N3, bond length of 2.205 (3) Å. The Fe1—O_water_ bond lengths [2.115 (2) and 2.066 (2) Å] are similar to the Fe1—O_carboxyl­ate_ bond lengths [2.131 (2) and 2.139 (2) Å].

The geometry of the sixfold coordinated metal atoms can best be described as a distorted octa­hedron, with atoms O1,N3,O1*W*,O3^i^ in the equatorial plane and atoms O2*W* and N1 in the apical positions with an O2*W*—Mn1—N1 bond angle of 163.62 (11) ° (Table 1 ▸), and an O2*W*—Fe1—N1 bond angle of 165.15 (10)° (Table 2 ▸). The coordinating pyridine ring (N3/C5–C9) and the carboxyl­ate group (O1/O2/C15) are inclined to the mean plane of the pyrazine ring by 18.57 (17) and 7.8 (4)°, respectively, in (I) and by 14.71 (16) and 7.4 (4)°, respectively, in (II). The non-coordinating pyridine ring (N4/C10–C14) and the second coordinating carboxyl­ate group (O3/O4/C16) are inclined to the mean plane of the pyrazine ring by 65.42 (16) and 80.64 (4)°, respectively, in (I) and by 64.59 (16) and 79.4 (4)°, respectively, in (II). In compound (I) the two pyridine rings are inclined to one another by 57.16 (18)°, very similar to the same dihedral angle in (II), *viz.* 57.28 (17)°.

## Supra­molecular features   

**Figure 4:**
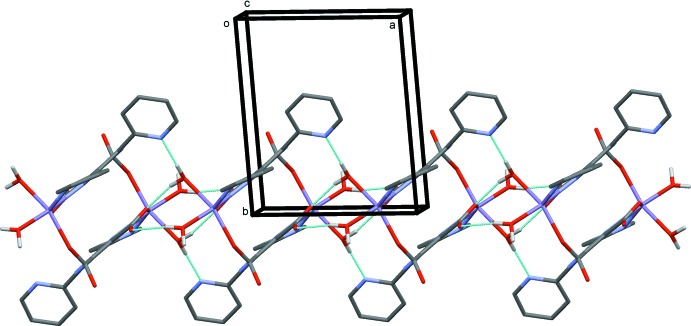
A view along the *c* axis of the chain of complexes propagating along the *a* axis direction. The hydrogen bonds are shown as dashed lines (see Table 3 ▸).

**Figure 5:**
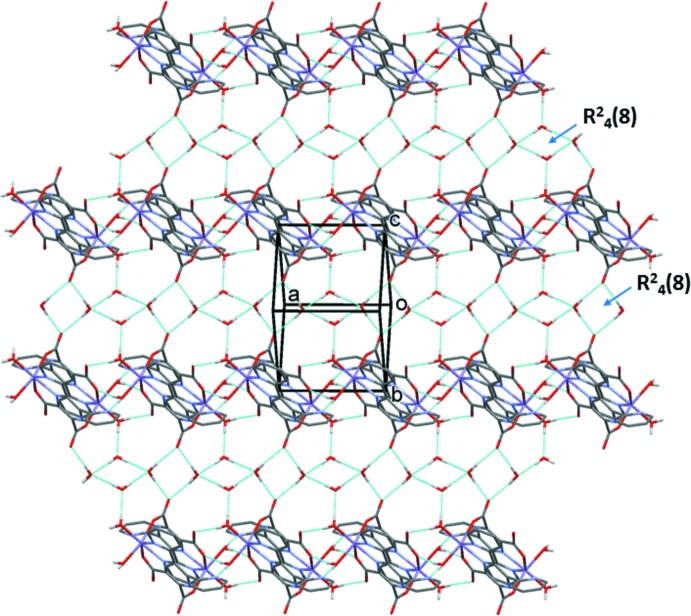
A view along the normal to the *bc* plane of the crystal packing of compound (I). The hydrogen bonds are shown as dashed lines (see Table 3 ▸), and the C-bound H atoms have been omitted for clarity.

**Figure 6:**
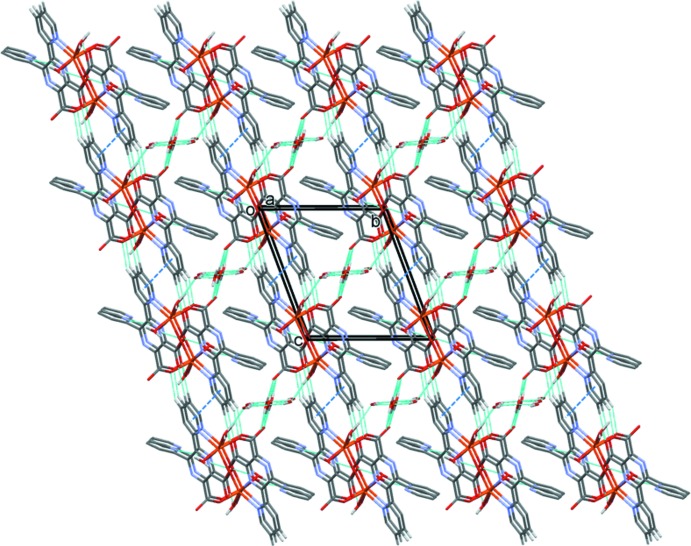
A view along the *a* axis of the crystal packing of compound (II), showing the hydrogen bonds as dashed lines (see Table 4 ▸). The offset π–π inter­actions are shown as dark-blue dashed lines and for clarity only the C-bound H atoms, H7 and H8, have been included.

Details of the hydrogen-bonding inter­actions in the crystals of both compounds, are given in Table 3 ▸ for (I) and Table 4 ▸ for (II). In the crystals of both compounds, the complexes are linked by O—H⋯O and O—H⋯N hydrogen bonds, involving the coordinating water mol­ecules (O1*W* and O2*W*), forming chains along [100]; illustrated in Fig. 4 ▸ for compound (I). The chains are linked by O—H⋯O hydrogen bonds involving the lattice water mol­ecules (O3*W* and O4*W*), forming layers parallel to the *bc* plane, as illustrated in Fig. 5 ▸ for compound (I). The lattice water mol­ecules are hydrogen bonded to themselves, forming chains that enclose two different 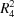
(8) ring motifs (Fig. 5 ▸). Pairs of C—H⋯O hydrogen bonds and offset π–π inter­actions, involving inversion-related coordinated pyridine rings [*Cg*⋯*Cg* ^ii^ = 3.671 (4) Å in (I), and 3.594 (2) Å in (II); *Cg* is the centroid of the ring N3/C5–C9; symmetry code: (ii) −*x* + 2, −*y* + 2, −*z* + 1], link the layers, forming a three-dimensional framework; illus­trated in Fig. 6 ▸ for compound (II).

## Database survey   

A search of the Cambridge Structural Database (CSD, Version 5.37, last update May 2016; Groom *et al.*, 2016 ▸) for the ligand **H_2_L**, and its dimethyl ester, gave eight hits. Some of these structures have been mentioned in the *Chemical context* above. In the case of (I) and (II), the ligand coordinates to the metal atom in a tridentate (*N,N,O*) and monodentate (*O*) manner. This coordination mode of **H_2_L** is the same as that observed in the Cd^II^ two-dimensional coordination polymer (Alfonso & Stoeckli-Evans, 2016 ▸). The pyridine rings and the carboxyl­ate groups are orientated with respect to the pyrazine ring in a very similar manner for all three compounds.

## Synthesis and crystallization   

The synthesis of the ligand 5,6-bis­(pyridin-2-yl)pyrazine-2,3-di­carb­oxy­lic acid (**H_2_L**) has been reported previously (Alfonso *et al.*, 2001 ▸).

**Synthesis of compound (I)**: **H_2_L** (64 mg, 0.20 mmol) was added in solid form to an aqueous solution (15 ml) of MnCl_2_·4H_2_O (45 mg, 0.20 mmol). The yellow solution immediately obtained was stirred for 10 min at room temperature, filtered and the filtrate allowed to slowly evaporate. After two weeks orange–yellow rod-like crystals were obtained. They were separated by filtration and dried in air (yield: 54 mg, 54.5%). Selected IR bands (KBr pellet, cm^−1^): ν 3226(*br*, *s*), 3080(*w*), 1636(*s*), 1598(*vs*), 1545(*w*), 1475(*m*), 1440(*m*), 1410(*w*), 1366(*s*), 1348(*s*), 1301(*w*), 1275(*w*), 1170(*m*), 1126(*m*), 1007(*w*), 954(*w*), 850(*w*), 790(*m*), 562(*m*).

**Synthesis of compound (II)**: A degassed aqueous solution (20 ml) of **H_2_L** (32 mg, 0.10 mmol) was treated with FeCl_2_·4H_2_O (20 mg, 0.10 mmol). The violet solution immediately obtained was stirred under N_2_ at 343 K for 1 h, filtered and the filtrate allowed to slowly evaporate. After two months deep-violet block-like crystals were obtained. They were separated by filtration and air dried (yield: 20 mg, 44.6%). Precipitation of small amounts of iron(III) hydroxide accompanied the formation of the crystals. Selected IR bands (KBr pellet, cm^−1^): ν 3477(*br*, *s*), 3291(*br*, *s*), 3078(*w*), 1640(*s*), 1593(*vs*), 1545(*w*), 1475(*m*), 1440(*m*), 1405(*w*), 1359(*m*), 1300(*w*), 1286(*w*), 1269(*w*), 1172(*m*), 1124(*m*), 1008(*w*), 954(*w*), 847(*w*), 789(*m*), 772(*w*), 677(*w*), 565(*m*), 549(*w*), 494(*m*) %.

## Refinement   

Crystal data, data collection and structure refinement details are summarized in Table 5 ▸. For both (I) and (II), the water H atoms were located in difference Fourier maps and refined with distance restraints: O—H = 0.84 (2) Å. The C-bound H atoms were included in calculated positions and treated as riding atoms: C—H = 0.93 Å for (I) and 0.94 Å for (II), with *U*_iso_(H) = 1.2*U*_eq_(C). Intensity data for (I) were collected at 293 K on a four-circle diffractometer. Only one equivalent of data was measured, hence *R*_int_ = 0, and as no suitable ψ-scans could be measured no absorption correction was applied. For compound (II), the data were collected at 223 K using a one-circle image-plate diffractometer with which it is not possible to measure 100% of the Ewald sphere, particularly for the triclinic system, hence a small cusp of data was inaccessible.

## Supplementary Material

Crystal structure: contains datablock(s) I, II, Global. DOI: 10.1107/S2056989016014055/pj2034sup1.cif

Structure factors: contains datablock(s) I. DOI: 10.1107/S2056989016014055/pj2034Isup2.hkl

Structure factors: contains datablock(s) II. DOI: 10.1107/S2056989016014055/pj2034IIsup3.hkl

CCDC references: 1502352, 1502351

Additional supporting information:  crystallographic information; 3D view; checkCIF report

## Figures and Tables

**Figure 2:**
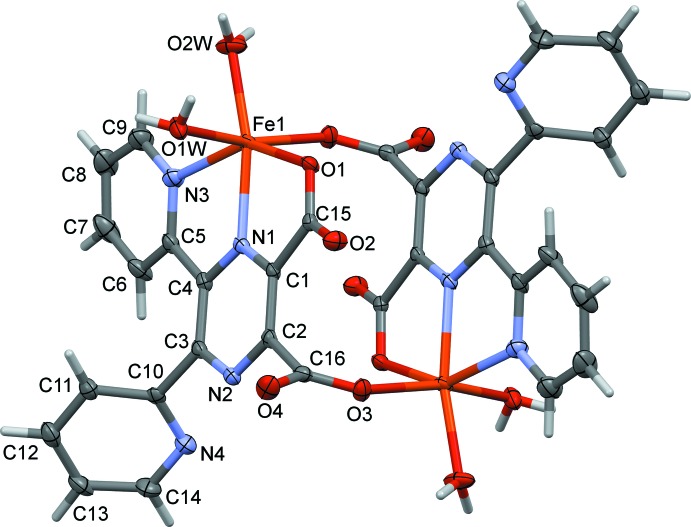
A view of the mol­ecular structure of compound (II), with atom labelling. Unlabelled atoms are related to the labelled atoms by inversion symmetry (−*x* + 2, −*y* + 2, −*z* + 2). Displacement ellipsoids are drawn at the 50% probability level. The solvate water molecules have been omitted for clarity.

**Table 1:** Selected geometric parameters (Å, °) for (I)

Mn1—O3^i^	2.139 (2)	Mn1—N3	2.311 (3)
Mn1—O1*W*	2.141 (3)	O1—C15	1.257 (4)
Mn1—O2*W*	2.148 (3)	O2—C15	1.243 (4)
Mn1—O1	2.228 (2)	O3—C16	1.254 (4)
Mn1—N1	2.242 (3)	O4—C16	1.239 (4)
			
O3^i^—Mn1—O1*W*	162.15 (10)	O2*W*—Mn1—N1	163.62 (11)
O3^i^—Mn1—O2*W*	85.00 (11)	O1—Mn1—N1	71.84 (8)
O1*W*—Mn1—O2*W*	86.92 (12)	O3^i^—Mn1—N3	100.80 (10)
O3^i^—Mn1—O1	89.80 (9)	O1*W*—Mn1—N3	95.57 (11)
O1*W*—Mn1—O1	81.62 (10)	O2*W*—Mn1—N3	93.71 (11)
O2*W*—Mn1—O1	124.11 (10)	O1—Mn1—N3	141.63 (9)
O3^i^—Mn1—N1	99.80 (10)	N1—Mn1—N3	70.05 (9)
O1*W*—Mn1—N1	92.39 (11)		

Symmetry code: (i) 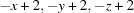.

**Table 2:** Selected geometric parameters (Å, °) for (II)

Fe1—O3^i^	2.105 (2)	Fe1—N3	2.205 (3)
Fe1—O1*W*	2.115 (2)	O1—C15	1.271 (4)
Fe1—O2*W*	2.066 (2)	O2—C15	1.229 (4)
Fe1—O1	2.131 (2)	O3—C16	1.251 (3)
Fe1—N1	2.126 (2)	O4—C16	1.240 (4)
			
O3^i^—Fe1—O1*W*	164.77 (9)	O2*W*—Fe1—N1	165.15 (10)
O3^i^—Fe1—O2*W*	85.22 (9)	N1—Fe1—O1	74.91 (9)
O1*W*—Fe1—O2*W*	87.43 (10)	O3^i^—Fe1—N3	99.60 (9)
O3^i^—Fe1—O1	89.65 (8)	O1*W*—Fe1—N3	94.03 (10)
O1*W*—Fe1—O1	82.38 (9)	O2*W*—Fe1—N3	92.45 (10)
O2*W*—Fe1—O1	119.49 (10)	O1—Fe1—N3	147.49 (9)
O3^i^—Fe1—N1	99.21 (9)	N1—Fe1—N3	72.87 (10)
O1*W*—Fe1—N1	91.27 (9)		

Symmetry code: (i) 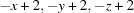.

**Table 3:** Hydrogen-bond geometry (Å, °) for (I)

*D*—H⋯*A*	*D*—H	H⋯*A*	*D*⋯*A*	*D*—H⋯*A*
O1*W*—H1*WA*⋯O1^ii^	0.83 (2)	1.89 (2)	2.710 (4)	168 (4)
O1*W*—H1*WB*⋯N4^iii^	0.84 (2)	1.91 (2)	2.750 (4)	179 (5)
O2*W*—H2*WA*⋯O2^ii^	0.84 (2)	1.91 (2)	2.743 (4)	170 (5)
O2*W*—H2*WB*⋯O3*W* ^iv^	0.83 (2)	1.92 (3)	2.710 (4)	159 (4)
O3*W*—H3*WA*⋯O4*W*	0.83 (2)	2.08 (2)	2.888 (5)	163 (5)
O3*W*—H3*WB*⋯O4*W* ^v^	0.85 (2)	2.07 (2)	2.906 (5)	169 (5)
O4*W*—H4*WA*⋯O4^vi^	0.84 (2)	2.16 (2)	3.000 (4)	175 (6)
O4*W*—H4*WB*⋯O4^vii^	0.84 (2)	1.95 (2)	2.793 (4)	176 (6)
C7—H7⋯O3^viii^	0.93	2.40	3.216 (5)	147
C8—H8⋯O2^viii^	0.93	2.54	3.452 (4)	167

Symmetry codes: (ii) 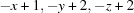; (iii) 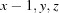; (iv) 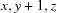; (v) 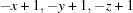; (vi) 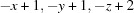; (vii) 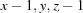; (viii) 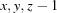.

**Table 4:** Hydrogen-bond geometry (Å, °) for (II)

*D*—H⋯*A*	*D*—H	H⋯*A*	*D*⋯*A*	*D*—H⋯*A*
O1*W*—H1*WA*⋯O1^ii^	0.84 (2)	1.93 (2)	2.728 (3)	160 (4)
O1*W*—H1*WB*⋯N4^iii^	0.83 (2)	1.94 (2)	2.752 (3)	165 (6)
O2*W*—H2*WA*⋯O2^ii^	0.87 (2)	1.83 (2)	2.694 (3)	172 (3)
O2*W*—H2*WB*⋯O3*W* ^iv^	0.84 (2)	1.87 (2)	2.686 (4)	165 (4)
O3*W*—H3*WA*⋯O4*W*	0.83 (2)	2.04 (2)	2.874 (4)	176 (6)
O3*W*—H3*WB*⋯O4*W* ^v^	0.84 (2)	2.03 (2)	2.866 (4)	171 (4)
O4*W*—H4*WA*⋯O4^vi^	0.84 (2)	2.12 (2)	2.957 (4)	172 (5)
O4*W*—H4*WB*⋯O4^vii^	0.82 (2)	1.96 (2)	2.784 (4)	178 (7)
C7—H7⋯O3^viii^	0.94	2.36	3.206 (5)	149
C8—H8⋯O2^viii^	0.94	2.57	3.477 (4)	162

Symmetry codes: (ii) 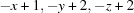; (iii) 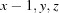; (iv) 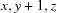; (v) 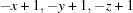; (vi) 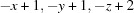; (vii) 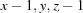; (viii) 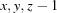.

**Table 5:** Experimental details

	(I)	(II)
Crystal data		
Chemical formula	[Mn_2_(C_16_H_8_N_4_O_4_)_2_(H_2_O)_4_]·4H_2_O	[Fe_2_(C_16_H_8_N_4_O_4_)_2_(H_2_O)_4_]·4H_2_O
*M*_r_	894.53	896.35
Crystal system, space group	Triclinic, *P* 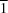	Triclinic, *P* 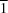
Temperature (K)	293	223
*a*, *b*, *c* (Å)	8.148 (7), 10.4408 (13), 11.5796 (11)	8.0933 (9), 10.3403 (11), 11.5679 (12)
α, β, γ (°)	70.527 (8), 84.232 (9), 84.849 (8)	69.500 (12), 83.593 (13), 84.238 (13)
*V* (Å^3^)	922.4 (8)	899.16 (18)
*Z*	1	1
Radiation type	Mo *K*α	Mo *K*α
μ (mm^−1^)	0.77	0.90
Crystal size (mm)	0.38 × 0.27 × 0.23	0.30 × 0.20 × 0.10

Data collection		
Diffractometer	Stoe–Siemens AED2, 4-circle	Stoe IPDS 1 image plate
Absorption correction	–	Multi-scan (*MULABS*; Spek, 2009 ▸)
*T*_min_, *T*_max_	–	0.805, 1.000
No. of measured, independent and observed [*I* > 2σ(*I*)] reflections	3244, 3244, 2689	6988, 3202, 2475
*R*_int_	0.000	0.063
(sin θ/λ)_max_ (Å^−1^)	0.595	0.611

Refinement		
*R*[*F* ^2^ > 2σ(*F* ^2^)], *wR*(*F* ^2^), *S*	0.045, 0.108, 1.11	0.046, 0.116, 0.97
No. of reflections	3244	3202
No. of parameters	294	294
No. of restraints	8	8
H-atom treatment	H atoms treated by a mixture of independent and constrained refinement	H atoms treated by a mixture of independent and constrained refinement
Δρ_max_, Δρ_min_ (e Å^−3^)	0.45, −0.40	0.59, −0.69

Computer programs: *STADI4 Software* (Stoe & Cie, 1997 ▸), *EXPOSE*, *CELL* and *INTEGRATE* in *IPDS-I* (Stoe & Cie, 2004 ▸), *X-RED* Software (Stoe & Cie, 1997 ▸), *SHELXS97* (Sheldrick, 2008 ▸), *SHELXL2014* (Sheldrick, 2015 ▸), *Mercury* (Macrae *et al.*, 2008 ▸), *PLATON* (Spek, 2009 ▸) and *publCIF* (Westrip, 2010 ▸).

## supplementary crystallographic information

### (I) Bis[µ-5,6-bis(pyridin-2-yl)pyrazine-2,3-dicarboxylato]-κ4N1,O2,N6:O3;κ4O3:N1,O2,N6-bis[diaquamanganese(II)] tetrahydrate Crystal data

**Table:**

[Mn_2_(C_16_H_8_N_4_O_4_)_2_(H_2_O)_4_]·4H_2_O	*Z* = 1
*M**_r_* = 894.53	*F*(000) = 458
Triclinic, *P*1	*D*_x_ = 1.610 Mg m^−^^3^
*a* = 8.148 (7) Å	Mo *K*α radiation, λ = 0.71073 Å
*b* = 10.4408 (13) Å	Cell parameters from 22 reflections
*c* = 11.5796 (11) Å	θ = 14.0–19.7°
α = 70.527 (8)°	µ = 0.77 mm^−^^1^
β = 84.232 (9)°	*T* = 293 K
γ = 84.849 (8)°	Block, yellow
*V* = 922.4 (8) Å^3^	0.38 × 0.27 × 0.23 mm

### (I) Bis[µ-5,6-bis(pyridin-2-yl)pyrazine-2,3-dicarboxylato]-κ4N1,O2,N6:O3;κ4O3:N1,O2,N6-bis[diaquamanganese(II)] tetrahydrate Data collection

**Table:**

Stoe-Siemens AED2, 4-circle diffractometer	*R*_int_ = 0.000
Radiation source: fine-focus sealed tube	θ_max_ = 25.0°, θ_min_ = 2.1°
Plane graphite monochromator	*h* = −9→9
ω/\2q scans	*k* = −11→12
3244 measured reflections	*l* = 0→13
3244 independent reflections	3 standard reflections every 60 min
2689 reflections with *I* > 2σ(*I*)	intensity decay: 2%

### (I) Bis[µ-5,6-bis(pyridin-2-yl)pyrazine-2,3-dicarboxylato]-κ4N1,O2,N6:O3;κ4O3:N1,O2,N6-bis[diaquamanganese(II)] tetrahydrate Refinement

**Table:**

Refinement on *F*^2^	Primary atom site location: structure-invariant direct methods
Least-squares matrix: full	Secondary atom site location: difference Fourier map
*R*[*F*^2^ > 2σ(*F*^2^)] = 0.045	Hydrogen site location: mixed
*wR*(*F*^2^) = 0.108	H atoms treated by a mixture of independent and constrained refinement
*S* = 1.11	*w* = 1/[σ^2^(*F*_o_^2^) + (0.039*P*)^2^ + 1.0007*P*] where *P* = (*F*_o_^2^ + 2*F*_c_^2^)/3
3244 reflections	(Δ/σ)_max_ < 0.001
294 parameters	Δρ_max_ = 0.45 e Å^−^^3^
8 restraints	Δρ_min_ = −0.40 e Å^−^^3^

### (I) Bis[µ-5,6-bis(pyridin-2-yl)pyrazine-2,3-dicarboxylato]-κ4N1,O2,N6:O3;κ4O3:N1,O2,N6-bis[diaquamanganese(II)] tetrahydrate Special details

**Table:**

Geometry. All esds (except the esd in the dihedral angle between two l.s. planes) are estimated using the full covariance matrix. The cell esds are taken into account individually in the estimation of esds in distances, angles and torsion angles; correlations between esds in cell parameters are only used when they are defined by crystal symmetry. An approximate (isotropic) treatment of cell esds is used for estimating esds involving l.s. planes.

### (I) Bis[µ-5,6-bis(pyridin-2-yl)pyrazine-2,3-dicarboxylato]-κ4N1,O2,N6:O3;κ4O3:N1,O2,N6-bis[diaquamanganese(II)] tetrahydrate Fractional atomic coordinates and isotropic or equivalent isotropic displacement parameters (Å^2^)

**Table:**

	*x*	*y*	*z*	*U*_iso_*/*U*_eq_	
Mn1	0.70864 (6)	0.99063 (5)	0.82620 (4)	0.02391 (16)	
N1	0.9248 (3)	0.8600 (3)	0.9156 (2)	0.0206 (6)	
N2	1.1681 (3)	0.6867 (3)	1.0396 (2)	0.0235 (6)	
N3	0.8377 (4)	0.8748 (3)	0.7001 (3)	0.0312 (7)	
N4	1.4257 (3)	0.6290 (3)	0.8621 (3)	0.0321 (7)	
O1	0.7117 (3)	1.0051 (2)	1.0136 (2)	0.0265 (5)	
O2	0.8025 (3)	0.9040 (3)	1.2007 (2)	0.0319 (6)	
O3	1.1805 (3)	0.8180 (2)	1.2502 (2)	0.0304 (5)	
O4	1.0455 (3)	0.6281 (3)	1.3049 (2)	0.0365 (6)	
O1W	0.5391 (3)	0.8379 (3)	0.9213 (3)	0.0357 (6)	
H1WA	0.455 (4)	0.875 (4)	0.946 (4)	0.051 (13)*	
H1WB	0.505 (5)	0.774 (3)	0.903 (4)	0.053 (14)*	
O2W	0.5185 (3)	1.0850 (3)	0.7038 (3)	0.0424 (7)	
H2WA	0.419 (3)	1.079 (5)	0.733 (4)	0.058 (15)*	
H2WB	0.523 (5)	1.166 (2)	0.659 (4)	0.052 (14)*	
C1	0.9384 (4)	0.8447 (3)	1.0332 (3)	0.0199 (6)	
C2	1.0656 (4)	0.7588 (3)	1.0956 (3)	0.0209 (7)	
C3	1.1502 (4)	0.6992 (3)	0.9223 (3)	0.0215 (7)	
C4	1.0271 (4)	0.7907 (3)	0.8571 (3)	0.0221 (7)	
C5	0.9896 (4)	0.8167 (3)	0.7265 (3)	0.0251 (7)	
C6	1.0980 (5)	0.7841 (4)	0.6389 (3)	0.0352 (9)	
H6	1.2053	0.7500	0.6567	0.042*	
C7	1.0440 (6)	0.8031 (4)	0.5245 (3)	0.0446 (10)	
H7	1.1148	0.7812	0.4647	0.053*	
C8	0.8856 (6)	0.8542 (4)	0.4996 (3)	0.0466 (11)	
H8	0.8455	0.8634	0.4247	0.056*	
C9	0.7872 (5)	0.8917 (4)	0.5885 (3)	0.0423 (10)	
H9	0.6815	0.9303	0.5706	0.051*	
C10	1.2647 (4)	0.6068 (3)	0.8723 (3)	0.0220 (7)	
C11	1.2058 (4)	0.5036 (3)	0.8415 (3)	0.0315 (8)	
H11	1.0930	0.4917	0.8485	0.038*	
C12	1.3179 (5)	0.4179 (4)	0.7999 (3)	0.0359 (9)	
H12	1.2817	0.3471	0.7790	0.043*	
C13	1.4837 (5)	0.4392 (4)	0.7900 (3)	0.0382 (9)	
H13	1.5615	0.3834	0.7621	0.046*	
C14	1.5315 (5)	0.5442 (4)	0.8220 (4)	0.0398 (9)	
H14	1.6438	0.5576	0.8156	0.048*	
C15	0.8067 (4)	0.9252 (3)	1.0883 (3)	0.0220 (7)	
C16	1.0965 (4)	0.7345 (3)	1.2291 (3)	0.0249 (7)	
O3W	0.5321 (5)	0.3184 (4)	0.5099 (3)	0.0592 (9)	
H3WA	0.441 (4)	0.361 (4)	0.496 (4)	0.063 (16)*	
H3WB	0.605 (5)	0.376 (4)	0.496 (5)	0.064 (17)*	
O4W	0.2287 (4)	0.4854 (4)	0.5048 (3)	0.0541 (8)	
H4WA	0.156 (5)	0.450 (5)	0.561 (4)	0.09 (2)*	
H4WB	0.177 (6)	0.528 (5)	0.443 (4)	0.09 (2)*	

### (I) Bis[µ-5,6-bis(pyridin-2-yl)pyrazine-2,3-dicarboxylato]-κ4N1,O2,N6:O3;κ4O3:N1,O2,N6-bis[diaquamanganese(II)] tetrahydrate Atomic displacement parameters (Å^2^)

**Table:**

	*U*^11^	*U*^22^	*U*^33^	*U*^12^	*U*^13^	*U*^23^
Mn1	0.0229 (3)	0.0281 (3)	0.0212 (3)	0.0047 (2)	−0.00536 (19)	−0.0092 (2)
N1	0.0206 (13)	0.0254 (14)	0.0170 (13)	0.0020 (11)	−0.0013 (10)	−0.0094 (11)
N2	0.0227 (14)	0.0255 (14)	0.0241 (14)	0.0032 (11)	−0.0052 (11)	−0.0108 (12)
N3	0.0327 (16)	0.0421 (18)	0.0229 (15)	0.0071 (13)	−0.0094 (12)	−0.0164 (13)
N4	0.0252 (15)	0.0384 (17)	0.0387 (17)	0.0029 (13)	−0.0029 (13)	−0.0216 (14)
O1	0.0291 (12)	0.0296 (12)	0.0214 (12)	0.0084 (10)	−0.0043 (10)	−0.0110 (10)
O2	0.0309 (13)	0.0458 (15)	0.0210 (12)	0.0088 (11)	−0.0020 (10)	−0.0164 (11)
O3	0.0360 (14)	0.0363 (14)	0.0222 (12)	−0.0046 (11)	−0.0076 (10)	−0.0117 (10)
O4	0.0466 (16)	0.0360 (15)	0.0234 (13)	−0.0105 (12)	−0.0068 (11)	−0.0020 (11)
O1W	0.0290 (14)	0.0340 (15)	0.0490 (17)	−0.0058 (12)	0.0061 (12)	−0.0218 (13)
O2W	0.0254 (15)	0.0521 (19)	0.0360 (16)	0.0046 (13)	−0.0067 (12)	0.0031 (14)
C1	0.0225 (16)	0.0194 (16)	0.0172 (15)	−0.0022 (13)	0.0000 (12)	−0.0054 (12)
C2	0.0200 (16)	0.0236 (16)	0.0206 (16)	−0.0034 (13)	−0.0028 (13)	−0.0083 (13)
C3	0.0216 (16)	0.0218 (16)	0.0220 (16)	−0.0013 (13)	−0.0027 (13)	−0.0082 (13)
C4	0.0243 (17)	0.0245 (17)	0.0190 (16)	0.0026 (13)	−0.0032 (13)	−0.0096 (13)
C5	0.0330 (19)	0.0270 (17)	0.0169 (16)	0.0049 (14)	−0.0045 (14)	−0.0101 (14)
C6	0.040 (2)	0.041 (2)	0.0242 (18)	0.0142 (17)	−0.0039 (15)	−0.0133 (16)
C7	0.071 (3)	0.044 (2)	0.0187 (18)	0.012 (2)	0.0017 (18)	−0.0150 (17)
C8	0.071 (3)	0.051 (3)	0.0219 (19)	0.011 (2)	−0.0142 (19)	−0.0184 (18)
C9	0.046 (2)	0.054 (3)	0.031 (2)	0.0112 (19)	−0.0190 (18)	−0.0183 (19)
C10	0.0229 (16)	0.0230 (16)	0.0201 (16)	0.0048 (13)	−0.0050 (13)	−0.0078 (13)
C11	0.0291 (18)	0.0278 (19)	0.038 (2)	−0.0011 (15)	−0.0004 (15)	−0.0122 (16)
C12	0.049 (2)	0.0256 (19)	0.036 (2)	0.0006 (17)	−0.0038 (17)	−0.0142 (16)
C13	0.041 (2)	0.039 (2)	0.038 (2)	0.0157 (17)	−0.0044 (17)	−0.0209 (18)
C14	0.0249 (19)	0.051 (2)	0.051 (2)	0.0086 (17)	−0.0060 (17)	−0.029 (2)
C15	0.0206 (16)	0.0254 (17)	0.0229 (17)	−0.0040 (13)	−0.0004 (13)	−0.0113 (14)
C16	0.0221 (17)	0.0307 (19)	0.0223 (17)	0.0063 (14)	−0.0058 (13)	−0.0100 (15)
O3W	0.052 (2)	0.050 (2)	0.062 (2)	0.0047 (18)	−0.0119 (17)	0.0001 (16)
O4W	0.0378 (17)	0.066 (2)	0.0458 (19)	0.0026 (15)	−0.0105 (15)	0.0000 (16)

### (I) Bis[µ-5,6-bis(pyridin-2-yl)pyrazine-2,3-dicarboxylato]-κ4N1,O2,N6:O3;κ4O3:N1,O2,N6-bis[diaquamanganese(II)] tetrahydrate Geometric parameters (Å, º)

**Table:**

Mn1—O3^i^	2.139 (2)	C2—C16	1.524 (4)
Mn1—O1W	2.141 (3)	C3—C4	1.410 (4)
Mn1—O2W	2.148 (3)	C3—C10	1.496 (4)
Mn1—O1	2.228 (2)	C4—C5	1.502 (4)
Mn1—N1	2.242 (3)	C5—C6	1.386 (5)
Mn1—N3	2.311 (3)	C6—C7	1.383 (5)
N1—C1	1.333 (4)	C6—H6	0.9300
N1—C4	1.335 (4)	C7—C8	1.372 (6)
N2—C2	1.337 (4)	C7—H7	0.9300
N2—C3	1.342 (4)	C8—C9	1.379 (6)
N3—C9	1.345 (4)	C8—H8	0.9300
N3—C5	1.346 (4)	C9—H9	0.9300
N4—C10	1.339 (4)	C10—C11	1.380 (5)
N4—C14	1.341 (4)	C11—C12	1.385 (5)
O1—C15	1.257 (4)	C11—H11	0.9300
O2—C15	1.243 (4)	C12—C13	1.376 (5)
O3—C16	1.254 (4)	C12—H12	0.9300
O3—Mn1^i^	2.139 (2)	C13—C14	1.367 (5)
O4—C16	1.239 (4)	C13—H13	0.9300
O1W—H1WA	0.831 (19)	C14—H14	0.9300
O1W—H1WB	0.839 (19)	O3W—H3WA	0.834 (19)
O2W—H2WA	0.841 (19)	O3W—H3WB	0.851 (19)
O2W—H2WB	0.833 (19)	O4W—H4WA	0.84 (2)
C1—C2	1.397 (4)	O4W—H4WB	0.84 (2)
C1—C15	1.524 (4)		
			
O3^i^—Mn1—O1W	162.15 (10)	N1—C4—C5	113.8 (3)
O3^i^—Mn1—O2W	85.00 (11)	C3—C4—C5	127.3 (3)
O1W—Mn1—O2W	86.92 (12)	N3—C5—C6	121.5 (3)
O3^i^—Mn1—O1	89.80 (9)	N3—C5—C4	114.0 (3)
O1W—Mn1—O1	81.62 (10)	C6—C5—C4	124.5 (3)
O2W—Mn1—O1	124.11 (10)	C7—C6—C5	118.9 (3)
O3^i^—Mn1—N1	99.80 (10)	C7—C6—H6	120.5
O1W—Mn1—N1	92.39 (11)	C5—C6—H6	120.5
O2W—Mn1—N1	163.62 (11)	C8—C7—C6	119.7 (3)
O1—Mn1—N1	71.84 (8)	C8—C7—H7	120.1
O3^i^—Mn1—N3	100.80 (10)	C6—C7—H7	120.1
O1W—Mn1—N3	95.57 (11)	C7—C8—C9	118.3 (3)
O2W—Mn1—N3	93.71 (11)	C7—C8—H8	120.8
O1—Mn1—N3	141.63 (9)	C9—C8—H8	120.8
N1—Mn1—N3	70.05 (9)	N3—C9—C8	122.8 (4)
C1—N1—C4	121.1 (3)	N3—C9—H9	118.6
C1—N1—Mn1	117.1 (2)	C8—C9—H9	118.6
C4—N1—Mn1	121.5 (2)	N4—C10—C11	122.8 (3)
C2—N2—C3	119.7 (3)	N4—C10—C3	116.1 (3)
C9—N3—C5	118.5 (3)	C11—C10—C3	121.1 (3)
C9—N3—Mn1	122.3 (2)	C10—C11—C12	118.7 (3)
C5—N3—Mn1	117.1 (2)	C10—C11—H11	120.7
C10—N4—C14	117.2 (3)	C12—C11—H11	120.7
C15—O1—Mn1	119.75 (19)	C13—C12—C11	119.0 (3)
C16—O3—Mn1^i^	145.3 (2)	C13—C12—H12	120.5
Mn1—O1W—H1WA	108 (3)	C11—C12—H12	120.5
Mn1—O1W—H1WB	132 (3)	C14—C13—C12	118.6 (3)
H1WA—O1W—H1WB	105 (4)	C14—C13—H13	120.7
Mn1—O2W—H2WA	118 (3)	C12—C13—H13	120.7
Mn1—O2W—H2WB	121 (3)	N4—C14—C13	123.7 (4)
H2WA—O2W—H2WB	104 (4)	N4—C14—H14	118.1
N1—C1—C2	119.7 (3)	C13—C14—H14	118.1
N1—C1—C15	114.6 (3)	O2—C15—O1	127.1 (3)
C2—C1—C15	125.7 (3)	O2—C15—C1	117.5 (3)
N2—C2—C1	120.2 (3)	O1—C15—C1	115.4 (3)
N2—C2—C16	114.9 (3)	O4—C16—O3	126.5 (3)
C1—C2—C16	124.9 (3)	O4—C16—C2	116.0 (3)
N2—C3—C4	120.3 (3)	O3—C16—C2	117.2 (3)
N2—C3—C10	114.7 (3)	H3WA—O3W—H3WB	108 (5)
C4—C3—C10	124.9 (3)	H4WA—O4W—H4WB	106 (5)
N1—C4—C3	118.8 (3)		
			
C4—N1—C1—C2	2.5 (5)	C5—C6—C7—C8	0.4 (6)
Mn1—N1—C1—C2	176.7 (2)	C6—C7—C8—C9	3.2 (6)
C4—N1—C1—C15	−176.9 (3)	C5—N3—C9—C8	−1.1 (6)
Mn1—N1—C1—C15	−2.7 (3)	Mn1—N3—C9—C8	161.9 (3)
C3—N2—C2—C1	1.2 (5)	C7—C8—C9—N3	−2.9 (7)
C3—N2—C2—C16	179.1 (3)	C14—N4—C10—C11	−1.2 (5)
N1—C1—C2—N2	−3.5 (5)	C14—N4—C10—C3	177.5 (3)
C15—C1—C2—N2	175.8 (3)	N2—C3—C10—N4	−65.6 (4)
N1—C1—C2—C16	178.9 (3)	C4—C3—C10—N4	116.5 (4)
C15—C1—C2—C16	−1.8 (5)	N2—C3—C10—C11	113.1 (3)
C2—N2—C3—C4	1.9 (5)	C4—C3—C10—C11	−64.8 (5)
C2—N2—C3—C10	−176.2 (3)	N4—C10—C11—C12	1.0 (5)
C1—N1—C4—C3	0.6 (5)	C3—C10—C11—C12	−177.6 (3)
Mn1—N1—C4—C3	−173.3 (2)	C10—C11—C12—C13	−0.4 (5)
C1—N1—C4—C5	177.8 (3)	C11—C12—C13—C14	0.2 (6)
Mn1—N1—C4—C5	3.9 (4)	C10—N4—C14—C13	0.9 (6)
N2—C3—C4—N1	−2.8 (5)	C12—C13—C14—N4	−0.4 (6)
C10—C3—C4—N1	175.0 (3)	Mn1—O1—C15—O2	−166.2 (3)
N2—C3—C4—C5	−179.7 (3)	Mn1—O1—C15—C1	12.6 (4)
C10—C3—C4—C5	−1.8 (5)	N1—C1—C15—O2	172.6 (3)
C9—N3—C5—C6	4.9 (5)	C2—C1—C15—O2	−6.7 (5)
Mn1—N3—C5—C6	−158.9 (3)	N1—C1—C15—O1	−6.3 (4)
C9—N3—C5—C4	−174.5 (3)	C2—C1—C15—O1	174.3 (3)
Mn1—N3—C5—C4	21.7 (4)	Mn1^i^—O3—C16—O4	−176.0 (3)
N1—C4—C5—N3	−16.8 (4)	Mn1^i^—O3—C16—C2	9.6 (5)
C3—C4—C5—N3	160.2 (3)	N2—C2—C16—O4	−77.6 (4)
N1—C4—C5—C6	163.8 (3)	C1—C2—C16—O4	100.2 (4)
C3—C4—C5—C6	−19.2 (6)	N2—C2—C16—O3	97.5 (4)
N3—C5—C6—C7	−4.6 (6)	C1—C2—C16—O3	−84.8 (4)
C4—C5—C6—C7	174.8 (3)		

Symmetry code: (i) −*x*+2, −*y*+2, −*z*+2.

### (I) Bis[µ-5,6-bis(pyridin-2-yl)pyrazine-2,3-dicarboxylato]-κ4N1,O2,N6:O3;κ4O3:N1,O2,N6-bis[diaquamanganese(II)] tetrahydrate Hydrogen-bond geometry (Å, º)

**Table:**

*D*—H···*A*	*D*—H	H···*A*	*D*···*A*	*D*—H···*A*
O1*W*—H1*WA*···O1^ii^	0.83 (2)	1.89 (2)	2.710 (4)	168 (4)
O1*W*—H1*WB*···N4^iii^	0.84 (2)	1.91 (2)	2.750 (4)	179 (5)
O2*W*—H2*WA*···O2^ii^	0.84 (2)	1.91 (2)	2.743 (4)	170 (5)
O2*W*—H2*WB*···O3*W*^iv^	0.83 (2)	1.92 (3)	2.710 (4)	159 (4)
O3*W*—H3*WA*···O4*W*	0.83 (2)	2.08 (2)	2.888 (5)	163 (5)
O3*W*—H3*WB*···O4*W*^v^	0.85 (2)	2.07 (2)	2.906 (5)	169 (5)
O4*W*—H4*WA*···O4^vi^	0.84 (2)	2.16 (2)	3.000 (4)	175 (6)
O4*W*—H4*WB*···O4^vii^	0.84 (2)	1.95 (2)	2.793 (4)	176 (6)
C7—H7···O3^viii^	0.93	2.40	3.216 (5)	147
C8—H8···O2^viii^	0.93	2.54	3.452 (4)	167

Symmetry codes: (ii) −*x*+1, −*y*+2, −*z*+2; (iii) *x*−1, *y*, *z*; (iv) *x*, *y*+1, *z*; (v) −*x*+1, −*y*+1, −*z*+1; (vi) −*x*+1, −*y*+1, −*z*+2; (vii) *x*−1, *y*, *z*−1; (viii) *x*, *y*, *z*−1.

### (II) Bis[µ-5,6-bis(pyridin-2-yl)pyrazine-2,3-dicarboxylato]-κ4N1,O2,N6:O3;κ4O3:N1,O2,N6-bis[diaquairon(II)] tetrahydrate Crystal data

**Table:**

[Fe_2_(C_16_H_8_N_4_O_4_)_2_(H_2_O)_4_]·4H_2_O	*Z* = 1
*M**_r_* = 896.35	*F*(000) = 460
Triclinic, *P*1	*D*_x_ = 1.655 Mg m^−^^3^
*a* = 8.0933 (9) Å	Mo *K*α radiation, λ = 0.71073 Å
*b* = 10.3403 (11) Å	Cell parameters from 5000 reflections
*c* = 11.5679 (12) Å	θ = 3.3–52.1°
α = 69.500 (12)°	µ = 0.90 mm^−^^1^
β = 83.593 (13)°	*T* = 223 K
γ = 84.238 (13)°	Block, dark-violet
*V* = 899.16 (18) Å^3^	0.30 × 0.20 × 0.10 mm

### (II) Bis[µ-5,6-bis(pyridin-2-yl)pyrazine-2,3-dicarboxylato]-κ4N1,O2,N6:O3;κ4O3:N1,O2,N6-bis[diaquairon(II)] tetrahydrate Data collection

**Table:**

Stoe IPDS 1 image plate diffractometer	3202 independent reflections
Radiation source: fine-focus sealed tube	2475 reflections with *I* > 2σ(*I*)
Plane graphite monochromator	*R*_int_ = 0.063
φ rotation scans	θ_max_ = 25.7°, θ_min_ = 2.1°
Absorption correction: multi-scan (MULABS; Spek, 2009)	*h* = −9→9
*T*_min_ = 0.805, *T*_max_ = 1.000	*k* = −12→12
6988 measured reflections	*l* = −14→14

### (II) Bis[µ-5,6-bis(pyridin-2-yl)pyrazine-2,3-dicarboxylato]-κ4N1,O2,N6:O3;κ4O3:N1,O2,N6-bis[diaquairon(II)] tetrahydrate Refinement

**Table:**

Refinement on *F*^2^	Primary atom site location: structure-invariant direct methods
Least-squares matrix: full	Secondary atom site location: difference Fourier map
*R*[*F*^2^ > 2σ(*F*^2^)] = 0.046	Hydrogen site location: mixed
*wR*(*F*^2^) = 0.116	H atoms treated by a mixture of independent and constrained refinement
*S* = 0.97	*w* = 1/[σ^2^(*F*_o_^2^) + (0.072*P*)^2^] where *P* = (*F*_o_^2^ + 2*F*_c_^2^)/3
3202 reflections	(Δ/σ)_max_ = 0.001
294 parameters	Δρ_max_ = 0.59 e Å^−^^3^
8 restraints	Δρ_min_ = −0.69 e Å^−^^3^

### (II) Bis[µ-5,6-bis(pyridin-2-yl)pyrazine-2,3-dicarboxylato]-κ4N1,O2,N6:O3;κ4O3:N1,O2,N6-bis[diaquairon(II)] tetrahydrate Special details

**Table:**

Geometry. All esds (except the esd in the dihedral angle between two l.s. planes) are estimated using the full covariance matrix. The cell esds are taken into account individually in the estimation of esds in distances, angles and torsion angles; correlations between esds in cell parameters are only used when they are defined by crystal symmetry. An approximate (isotropic) treatment of cell esds is used for estimating esds involving l.s. planes.

### (II) Bis[µ-5,6-bis(pyridin-2-yl)pyrazine-2,3-dicarboxylato]-κ4N1,O2,N6:O3;κ4O3:N1,O2,N6-bis[diaquairon(II)] tetrahydrate Fractional atomic coordinates and isotropic or equivalent isotropic displacement parameters (Å^2^)

**Table:**

	*x*	*y*	*z*	*U*_iso_*/*U*_eq_	
Fe1	0.71296 (5)	0.99041 (4)	0.82831 (4)	0.01544 (16)	
N1	0.9194 (3)	0.8624 (2)	0.9131 (2)	0.0142 (5)	
N2	1.1679 (3)	0.6879 (2)	1.0372 (2)	0.0154 (6)	
N3	0.8286 (3)	0.8855 (3)	0.7002 (3)	0.0202 (6)	
N4	1.4267 (3)	0.6299 (3)	0.8589 (3)	0.0226 (6)	
O1	0.7040 (3)	1.00868 (19)	1.0068 (2)	0.0165 (5)	
O2	0.7957 (3)	0.9106 (2)	1.1961 (2)	0.0211 (5)	
O3	1.1738 (3)	0.8201 (2)	1.2494 (2)	0.0194 (5)	
O4	1.0447 (3)	0.6252 (2)	1.3039 (2)	0.0245 (5)	
O1W	0.5488 (3)	0.8329 (2)	0.9222 (2)	0.0225 (5)	
H1WA	0.463 (3)	0.863 (4)	0.955 (4)	0.040 (12)*	
H1WB	0.508 (6)	0.784 (5)	0.891 (5)	0.078 (18)*	
O2W	0.5259 (3)	1.0878 (2)	0.7138 (3)	0.0277 (6)	
H2WA	0.423 (3)	1.080 (4)	0.744 (3)	0.028 (10)*	
H2WB	0.547 (5)	1.160 (3)	0.654 (3)	0.030 (11)*	
C1	0.9344 (3)	0.8476 (3)	1.0307 (3)	0.0123 (6)	
C2	1.0637 (4)	0.7604 (3)	1.0931 (3)	0.0137 (6)	
C3	1.1487 (4)	0.7011 (3)	0.9199 (3)	0.0151 (7)	
C4	1.0221 (4)	0.7921 (3)	0.8542 (3)	0.0139 (6)	
C5	0.9805 (4)	0.8186 (3)	0.7251 (3)	0.0182 (7)	
C6	1.0827 (4)	0.7811 (3)	0.6371 (3)	0.0254 (8)	
H6	1.1908	0.7411	0.6540	0.031*	
C7	1.0240 (5)	0.8032 (3)	0.5224 (4)	0.0320 (9)	
H7	1.0915	0.7775	0.4614	0.038*	
C8	0.8663 (5)	0.8629 (4)	0.4998 (4)	0.0326 (9)	
H8	0.8218	0.8744	0.4249	0.039*	
C9	0.7745 (5)	0.9057 (3)	0.5888 (3)	0.0292 (8)	
H9	0.6690	0.9512	0.5710	0.035*	
C10	1.2661 (4)	0.6075 (3)	0.8702 (3)	0.0150 (6)	
C11	1.2068 (4)	0.5027 (3)	0.8409 (3)	0.0212 (7)	
H11	1.0922	0.4899	0.8496	0.025*	
C12	1.3217 (4)	0.4167 (3)	0.7982 (3)	0.0252 (8)	
H12	1.2857	0.3450	0.7768	0.030*	
C13	1.4882 (4)	0.4381 (3)	0.7877 (3)	0.0268 (8)	
H13	1.5685	0.3808	0.7601	0.032*	
C14	1.5346 (4)	0.5459 (3)	0.8187 (4)	0.0282 (8)	
H14	1.6486	0.5608	0.8111	0.034*	
C15	0.8008 (3)	0.9290 (3)	1.0849 (3)	0.0137 (6)	
C16	1.0942 (4)	0.7348 (3)	1.2273 (3)	0.0162 (7)	
O3W	0.5377 (4)	0.3176 (3)	0.5121 (3)	0.0389 (7)	
H3WA	0.448 (4)	0.365 (5)	0.507 (6)	0.08 (2)*	
H3WB	0.611 (4)	0.374 (4)	0.500 (4)	0.044 (13)*	
O4W	0.2296 (3)	0.4837 (3)	0.5051 (3)	0.0351 (6)	
H4WA	0.159 (5)	0.448 (4)	0.564 (3)	0.050 (14)*	
H4WB	0.177 (7)	0.527 (6)	0.445 (4)	0.10 (2)*	

### (II) Bis[µ-5,6-bis(pyridin-2-yl)pyrazine-2,3-dicarboxylato]-κ4N1,O2,N6:O3;κ4O3:N1,O2,N6-bis[diaquairon(II)] tetrahydrate Atomic displacement parameters (Å^2^)

**Table:**

	*U*^11^	*U*^22^	*U*^33^	*U*^12^	*U*^13^	*U*^23^
Fe1	0.0188 (3)	0.0121 (2)	0.0149 (3)	0.00398 (16)	−0.00588 (18)	−0.00385 (17)
N1	0.0159 (12)	0.0098 (11)	0.0163 (16)	0.0009 (9)	−0.0017 (11)	−0.0042 (10)
N2	0.0175 (13)	0.0114 (11)	0.0181 (16)	0.0014 (9)	−0.0042 (11)	−0.0058 (11)
N3	0.0237 (14)	0.0199 (13)	0.0170 (17)	0.0032 (11)	−0.0073 (12)	−0.0059 (12)
N4	0.0202 (14)	0.0204 (13)	0.0297 (19)	0.0033 (11)	−0.0044 (13)	−0.0124 (12)
O1	0.0200 (11)	0.0132 (9)	0.0166 (13)	0.0053 (8)	−0.0049 (9)	−0.0062 (9)
O2	0.0211 (11)	0.0273 (11)	0.0154 (14)	0.0050 (9)	−0.0034 (10)	−0.0093 (10)
O3	0.0269 (12)	0.0160 (10)	0.0155 (13)	−0.0035 (9)	−0.0053 (10)	−0.0042 (9)
O4	0.0328 (13)	0.0183 (11)	0.0173 (14)	−0.0067 (9)	−0.0059 (11)	0.0028 (10)
O1W	0.0193 (12)	0.0168 (11)	0.0338 (16)	−0.0020 (9)	0.0023 (11)	−0.0128 (11)
O2W	0.0184 (13)	0.0250 (12)	0.0284 (17)	0.0022 (10)	−0.0067 (11)	0.0052 (11)
C1	0.0154 (14)	0.0083 (12)	0.0130 (18)	−0.0002 (11)	−0.0014 (13)	−0.0035 (12)
C2	0.0173 (15)	0.0090 (12)	0.0143 (18)	−0.0028 (11)	−0.0015 (13)	−0.0027 (12)
C3	0.0161 (15)	0.0096 (13)	0.020 (2)	−0.0005 (11)	−0.0012 (13)	−0.0055 (12)
C4	0.0168 (15)	0.0111 (13)	0.0147 (18)	−0.0010 (11)	−0.0004 (13)	−0.0058 (12)
C5	0.0247 (16)	0.0110 (13)	0.018 (2)	0.0022 (12)	−0.0030 (14)	−0.0048 (13)
C6	0.0363 (19)	0.0189 (15)	0.020 (2)	0.0123 (14)	−0.0055 (16)	−0.0077 (14)
C7	0.052 (2)	0.0223 (16)	0.019 (2)	0.0090 (16)	−0.0002 (18)	−0.0073 (15)
C8	0.053 (2)	0.0293 (18)	0.018 (2)	0.0063 (16)	−0.0142 (18)	−0.0112 (16)
C9	0.037 (2)	0.0278 (17)	0.024 (2)	0.0082 (15)	−0.0119 (17)	−0.0110 (16)
C10	0.0209 (15)	0.0111 (13)	0.0108 (18)	0.0028 (11)	−0.0054 (13)	−0.0007 (12)
C11	0.0232 (16)	0.0131 (14)	0.027 (2)	0.0000 (12)	−0.0036 (15)	−0.0058 (14)
C12	0.0371 (19)	0.0139 (14)	0.025 (2)	0.0012 (13)	−0.0041 (16)	−0.0069 (14)
C13	0.0325 (19)	0.0201 (15)	0.027 (2)	0.0112 (14)	−0.0035 (16)	−0.0102 (15)
C14	0.0190 (17)	0.0296 (17)	0.038 (3)	0.0072 (13)	−0.0039 (16)	−0.0166 (17)
C15	0.0140 (14)	0.0110 (13)	0.018 (2)	−0.0007 (11)	−0.0031 (13)	−0.0067 (12)
C16	0.0159 (15)	0.0153 (14)	0.0166 (19)	0.0044 (12)	−0.0041 (13)	−0.0050 (13)
O3W	0.0379 (17)	0.0250 (13)	0.042 (2)	0.0034 (12)	−0.0074 (14)	0.0028 (12)
O4W	0.0258 (14)	0.0379 (15)	0.0308 (19)	−0.0003 (11)	−0.0060 (13)	0.0024 (13)

### (II) Bis[µ-5,6-bis(pyridin-2-yl)pyrazine-2,3-dicarboxylato]-κ4N1,O2,N6:O3;κ4O3:N1,O2,N6-bis[diaquairon(II)] tetrahydrate Geometric parameters (Å, º)

**Table:**

Fe1—O3^i^	2.105 (2)	C2—C16	1.525 (4)
Fe1—O1W	2.115 (2)	C3—C4	1.411 (4)
Fe1—O2W	2.066 (2)	C3—C10	1.502 (4)
Fe1—O1	2.131 (2)	C4—C5	1.492 (4)
Fe1—N1	2.126 (2)	C5—C6	1.377 (5)
Fe1—N3	2.205 (3)	C6—C7	1.394 (5)
N1—C1	1.333 (4)	C6—H6	0.9400
N1—C4	1.335 (4)	C7—C8	1.372 (5)
N2—C2	1.335 (4)	C7—H7	0.9400
N2—C3	1.340 (4)	C8—C9	1.374 (5)
N3—C9	1.347 (4)	C8—H8	0.9400
N3—C5	1.357 (4)	C9—H9	0.9400
N4—C10	1.327 (4)	C10—C11	1.385 (4)
N4—C14	1.331 (4)	C11—C12	1.391 (5)
O1—C15	1.271 (4)	C11—H11	0.9400
O2—C15	1.229 (4)	C12—C13	1.373 (5)
O3—C16	1.251 (3)	C12—H12	0.9400
O3—Fe1^i^	2.105 (2)	C13—C14	1.381 (5)
O4—C16	1.240 (4)	C13—H13	0.9400
O1W—H1WA	0.836 (19)	C14—H14	0.9400
O1W—H1WB	0.83 (2)	O3W—H3WA	0.83 (2)
O2W—H2WA	0.867 (19)	O3W—H3WB	0.843 (19)
O2W—H2WB	0.840 (19)	O4W—H4WA	0.842 (19)
C1—C2	1.398 (4)	O4W—H4WB	0.82 (2)
C1—C15	1.519 (4)		
			
O3^i^—Fe1—O1W	164.77 (9)	N1—C4—C5	113.3 (2)
O3^i^—Fe1—O2W	85.22 (9)	C3—C4—C5	128.5 (3)
O1W—Fe1—O2W	87.43 (10)	N3—C5—C6	121.7 (3)
O3^i^—Fe1—O1	89.65 (8)	N3—C5—C4	113.3 (3)
O1W—Fe1—O1	82.38 (9)	C6—C5—C4	125.0 (3)
O2W—Fe1—O1	119.49 (10)	C5—C6—C7	119.2 (3)
O3^i^—Fe1—N1	99.21 (9)	C5—C6—H6	120.4
O1W—Fe1—N1	91.27 (9)	C7—C6—H6	120.4
O2W—Fe1—N1	165.15 (10)	C8—C7—C6	119.1 (3)
N1—Fe1—O1	74.91 (9)	C8—C7—H7	120.4
O3^i^—Fe1—N3	99.60 (9)	C6—C7—H7	120.4
O1W—Fe1—N3	94.03 (10)	C7—C8—C9	118.7 (3)
O2W—Fe1—N3	92.45 (10)	C7—C8—H8	120.6
O1—Fe1—N3	147.49 (9)	C9—C8—H8	120.6
N1—Fe1—N3	72.87 (10)	N3—C9—C8	123.1 (3)
C1—N1—C4	121.5 (2)	N3—C9—H9	118.4
C1—N1—Fe1	116.87 (19)	C8—C9—H9	118.4
C4—N1—Fe1	121.4 (2)	N4—C10—C11	123.0 (3)
C2—N2—C3	119.3 (2)	N4—C10—C3	116.3 (2)
C9—N3—C5	117.9 (3)	C11—C10—C3	120.7 (3)
C9—N3—Fe1	124.0 (2)	C10—C11—C12	118.2 (3)
C5—N3—Fe1	116.9 (2)	C10—C11—H11	120.9
C10—N4—C14	117.9 (3)	C12—C11—H11	120.9
C15—O1—Fe1	118.95 (19)	C13—C12—C11	119.1 (3)
C16—O3—Fe1^i^	144.9 (2)	C13—C12—H12	120.4
Fe1—O1W—H1WA	112 (3)	C11—C12—H12	120.4
Fe1—O1W—H1WB	126 (4)	C12—C13—C14	118.2 (3)
H1WA—O1W—H1WB	101 (4)	C12—C13—H13	120.9
Fe1—O2W—H2WA	119 (3)	C14—C13—H13	120.9
Fe1—O2W—H2WB	118 (3)	N4—C14—C13	123.5 (3)
H2WA—O2W—H2WB	117 (4)	N4—C14—H14	118.2
N1—C1—C2	119.6 (3)	C13—C14—H14	118.2
N1—C1—C15	114.3 (2)	O2—C15—O1	127.5 (3)
C2—C1—C15	126.1 (3)	O2—C15—C1	118.4 (2)
N2—C2—C1	120.4 (3)	O1—C15—C1	114.0 (3)
N2—C2—C16	115.0 (2)	O4—C16—O3	126.2 (3)
C1—C2—C16	124.6 (3)	O4—C16—C2	115.5 (2)
N2—C3—C4	121.1 (3)	O3—C16—C2	118.2 (3)
N2—C3—C10	114.3 (2)	H3WA—O3W—H3WB	105 (4)
C4—C3—C10	124.6 (3)	H4WA—O4W—H4WB	107 (5)
N1—C4—C3	118.1 (3)		
			
C4—N1—C1—C2	2.2 (4)	C5—C6—C7—C8	0.6 (5)
Fe1—N1—C1—C2	177.48 (19)	C6—C7—C8—C9	3.4 (5)
C4—N1—C1—C15	−176.6 (2)	C5—N3—C9—C8	−0.6 (5)
Fe1—N1—C1—C15	−1.3 (3)	Fe1—N3—C9—C8	166.1 (3)
C3—N2—C2—C1	1.0 (4)	C7—C8—C9—N3	−3.5 (6)
C3—N2—C2—C16	178.6 (2)	C14—N4—C10—C11	−0.8 (5)
N1—C1—C2—N2	−2.7 (4)	C14—N4—C10—C3	177.9 (3)
C15—C1—C2—N2	176.0 (2)	N2—C3—C10—N4	−65.1 (4)
N1—C1—C2—C16	−180.0 (2)	C4—C3—C10—N4	117.8 (3)
C15—C1—C2—C16	−1.4 (4)	N2—C3—C10—C11	113.7 (3)
C2—N2—C3—C4	1.1 (4)	C4—C3—C10—C11	−63.5 (4)
C2—N2—C3—C10	−176.2 (2)	N4—C10—C11—C12	0.3 (5)
C1—N1—C4—C3	−0.2 (4)	C3—C10—C11—C12	−178.3 (3)
Fe1—N1—C4—C3	−175.24 (19)	C10—C11—C12—C13	0.5 (5)
C1—N1—C4—C5	177.6 (2)	C11—C12—C13—C14	−0.8 (5)
Fe1—N1—C4—C5	2.6 (3)	C10—N4—C14—C13	0.4 (5)
N2—C3—C4—N1	−1.5 (4)	C12—C13—C14—N4	0.3 (6)
C10—C3—C4—N1	175.5 (3)	Fe1—O1—C15—O2	−168.1 (2)
N2—C3—C4—C5	−178.9 (3)	Fe1—O1—C15—C1	10.8 (3)
C10—C3—C4—C5	−2.0 (5)	N1—C1—C15—O2	172.9 (2)
C9—N3—C5—C6	4.8 (4)	C2—C1—C15—O2	−5.8 (4)
Fe1—N3—C5—C6	−162.8 (2)	N1—C1—C15—O1	−6.1 (3)
C9—N3—C5—C4	−175.5 (3)	C2—C1—C15—O1	175.2 (3)
Fe1—N3—C5—C4	16.9 (3)	Fe1^i^—O3—C16—O4	180.0 (2)
N1—C4—C5—N3	−12.7 (4)	Fe1^i^—O3—C16—C2	4.6 (5)
C3—C4—C5—N3	164.8 (3)	N2—C2—C16—O4	−76.7 (3)
N1—C4—C5—C6	167.0 (3)	C1—C2—C16—O4	100.8 (3)
C3—C4—C5—C6	−15.4 (5)	N2—C2—C16—O3	99.2 (3)
N3—C5—C6—C7	−4.8 (5)	C1—C2—C16—O3	−83.4 (4)
C4—C5—C6—C7	175.5 (3)		

Symmetry code: (i) −*x*+2, −*y*+2, −*z*+2.

### (II) Bis[µ-5,6-bis(pyridin-2-yl)pyrazine-2,3-dicarboxylato]-κ4N1,O2,N6:O3;κ4O3:N1,O2,N6-bis[diaquairon(II)] tetrahydrate Hydrogen-bond geometry (Å, º)

**Table:**

*D*—H···*A*	*D*—H	H···*A*	*D*···*A*	*D*—H···*A*
O1*W*—H1*WA*···O1^ii^	0.84 (2)	1.93 (2)	2.728 (3)	160 (4)
O1*W*—H1*WB*···N4^iii^	0.83 (2)	1.94 (2)	2.752 (3)	165 (6)
O2*W*—H2*WA*···O2^ii^	0.87 (2)	1.83 (2)	2.694 (3)	172 (3)
O2*W*—H2*WB*···O3*W*^iv^	0.84 (2)	1.87 (2)	2.686 (4)	165 (4)
O3*W*—H3*WA*···O4*W*	0.83 (2)	2.04 (2)	2.874 (4)	176 (6)
O3*W*—H3*WB*···O4*W*^v^	0.84 (2)	2.03 (2)	2.866 (4)	171 (4)
O4*W*—H4*WA*···O4^vi^	0.84 (2)	2.12 (2)	2.957 (4)	172 (5)
O4*W*—H4*WB*···O4^vii^	0.82 (2)	1.96 (2)	2.784 (4)	178 (7)
C7—H7···O3^viii^	0.94	2.36	3.206 (5)	149
C8—H8···O2^viii^	0.94	2.57	3.477 (4)	162

Symmetry codes: (ii) −*x*+1, −*y*+2, −*z*+2; (iii) *x*−1, *y*, *z*; (iv) *x*, *y*+1, *z*; (v) −*x*+1, −*y*+1, −*z*+1; (vi) −*x*+1, −*y*+1, −*z*+2; (vii) *x*−1, *y*, *z*−1; (viii) *x*, *y*, *z*−1.
